# Single-cell transcriptomics identifies a putative perivascular macrophage–endothelial MIF–ACKR3 axis associated with endothelial activation in acute traumatic brain injury

**DOI:** 10.3389/fncel.2026.1863509

**Published:** 2026-08-18

**Authors:** Yanya Lin, Chengda Lin, Jianhui Chen, Shijun Chen, Jianhuang Huang, Jianxiong Hu

**Affiliations:** 1 The Graduate School of Fujian Medical University, Fuzhou, Fujian, China; 2 Department of Critical Care Medicine, Affiliated Hospital of Putian University, Putian, Fujian, China; 3 Department of Neurosurgery, Putian 95 Hospital of China RongTong Medical and Health Group, Putian, Fujian, China; 4 Department of Neurosurgery, Affiliated Hospital of Putian University, Putian, Fujian, China

**Keywords:** ACKR3, blood–brain barrier, endothelial activation, MIF, neurovascular unit, perivascular macrophages, traumatic brain injury

## Abstract

**Background:**

Blood–brain barrier (BBB) disruption is a hallmark of acute traumatic brain injury (TBI), yet the immune–vascular mechanisms underlying endothelial dysfunction remain incompletely understood. Perivascular macrophages (PVMs) are strategically positioned to modulate cerebrovascular homeostasis, but their role in acute post-traumatic endothelial activation has not been systematically characterized.

**Methods:**

We re-analyzed a publicly available mouse single-cell RNA sequencing (scRNA-seq) dataset (GSE290150, 24 h post-TBI) to characterize the landscape of immune and vascular cell populations and to infer intercellular communication. Bone marrow–derived macrophages (BMDMs) served as a PVM surrogate model, and bEnd.3 cerebral endothelial cells were used for *in vitro* validation of key signaling pathways.

**Results:**

Single-cell analysis revealed concurrent expansion of PVM-like cells and activated endothelial cells (Activated ECs) in the TBI brain, with CellChat-predicted enhancement of MIF–ACKR3 signaling from PVM-like cells toward Activated ECs. Inflammatory stimulation of BMDMs significantly increased MIF expression and secretion in a STAT3-dependent manner. Macrophage-conditioned medium induced endothelial activation–associated molecular changes, including upregulation of Angpt2 and Adm and downregulation of tight junction genes Claudin5 and Tjp1; these effects were attenuated by MIF inhibitor ISO-1 or ACKR3 antagonist CCX771. Recombinant MIF dose-dependently reproduced these changes, which were substantially abrogated by ACKR3 pharmacological blockade or siRNA knockdown. STAT3 inhibition further suppressed rMIF-induced endothelial transcriptional responses.

**Conclusion:**

These findings provide transcriptomic and *in vitro* evidence supporting a PVM-derived MIF–ACKR3–STAT3 signaling axis associated with endothelial activation–related molecular changes in acute TBI. Direct functional effects on BBB integrity and *in vivo* relevance require further validation. This candidate axis highlights perivascular immune–vascular crosstalk as a potential avenue for therapeutic investigation.

## 1 Introduction

Traumatic brain injury (TBI) is a leading cause of death and long-term disability worldwide, imposing a profound burden on patients, families, and healthcare systems globally (Maas et al., 2017). The pathophysiology of TBI is conventionally framed as a biphasic process: an immediate primary mechanical insult that precipitates a complex, protracted secondary injury cascade encompassing neuroinflammation, oxidative stress, metabolic dysfunction, excitotoxicity, and cerebrovascular compromise (Ng and Lee, 2019). Animal models of TBI, including the controlled cortical impact (CCI) paradigm, have provided important mechanistic insights into these secondary injury processes (Xiong et al., 2013). Among these, disruption of blood–brain barrier (BBB) integrity is recognized as a central event that amplifies cerebral edema, facilitates inflammatory cell infiltration into the parenchyma, and contributes to progressive neuronal damage (Daneman and Prat, 2015; Sun et al., 2025).

The BBB is maintained by a highly specialized neurovascular unit (NVU) comprising cerebral microvascular endothelial cells, pericytes, astrocytes, neurons, and the basement membrane, all acting in concert to regulate selective permeability (Iadecola, 2017). Tight junction proteins—including Claudin-5, Occludin, and ZO-1 (encoded by Tjp1)—are fundamental determinants of paracellular barrier properties, and their transcriptional downregulation is closely associated with acute BBB compromise following TBI (Greene et al., 2019; Shlosberg et al., 2010). The 24–72 h post-injury window is particularly critical for BBB disruption, during which endothelial cells undergo reactive reprogramming characterized by upregulation of adhesion molecules, pro-inflammatory mediators, and vascular remodeling genes (Chodobski et al., 2011). Identifying the upstream immune signals that drive this endothelial state transition remains an important unresolved question with direct therapeutic implications.

Perivascular macrophages (PVMs) constitute a specialized subset of border-associated macrophages (BAMs) that reside within the perivascular Virchow–Robin spaces of cerebral blood vessels, developmentally and functionally distinct from parenchymal microglia (Kierdorf et al., 2019; Lapenna et al., 2018). Under homeostatic conditions, PVMs participate in immune surveillance, regulation of vascular tone, and facilitation of metabolic waste clearance via the glymphatic system (Drieu et al., 2022). Emerging evidence implicates PVMs in neurovascular pathology across multiple disease contexts, including hypertension-associated cognitive dysfunction (Faraco et al., 2016), amyloid clearance in cerebral amyloid angiopathy (Hawkes and McLaurin, 2009), and dynamic responses to ischemic vascular injury (Pedragosa et al., 2018). However, whether PVMs actively contribute to acute endothelial activation and BBB injury in TBI—and through which molecular mechanisms—has not been systematically investigated.

Macrophage migration inhibitory factor (MIF) is a pleiotropic pro-inflammatory cytokine constitutively expressed by macrophages, monocytes, endothelial cells, neurons, and other cell types (Calandra and Roger, 2003). MIF exerts its biological effects through multiple receptor pathways, including the CD74/CD44 complex (Leng et al., 2003) and the atypical chemokine receptor ACKR3 (also designated CXCR7), which has been identified as a functional non-cognate binding receptor for MIF capable of mediating intracellular signaling (Bernhagen et al., 2007). ACKR3 additionally functions as a scavenger receptor for multiple chemokine ligands including CXCL12 and CXCL11 (Naumann et al., 2010), and has been implicated in vascular inflammation, angiogenesis, and endothelial cell survival (Hattermann et al., 2010; Sánchez-Martín et al., 2011). Clinically, elevated serum MIF levels have been documented in TBI patients and correlate with injury severity and neurological prognosis (Yang et al., 2017), yet the primary cellular source of MIF within the injured brain parenchyma, its specific receptor targets at the cerebrovascular interface, and its potential contribution to endothelial injury and barrier dysfunction remain to be established. To our knowledge, prior studies have not combined single-cell interrogation of the acute TBI neurovascular compartment with mechanistic testing of a macrophage-to-endothelial MIF receptor axis. Most existing TBI studies implicate MIF at the systemic biomarker level, whereas studies in cardiovascular biology define MIF receptor signaling largely outside the acute brain injury context. The specific unresolved question addressed here is whether an acute TBI-expanded PVM-like population is positioned to signal to activated cerebral endothelium via an MIF–ACKR3 pathway.

Single-cell RNA sequencing (scRNA-seq) has transformed our ability to characterize cellular heterogeneity, identify rare cell subpopulations, and infer intercellular communication networks in complex tissues (Papalexi and Satija, 2018; Zeisel et al., 2015). Computational tools such as CellChat enable systematic inference of candidate ligand–receptor communication networks from single-cell transcriptomic data (Jin et al., 2021), providing hypothesis-generating frameworks for subsequent experimental validation. In the present study, we performed systematic re-analysis of a published scRNA-seq dataset from mouse brain tissue 24 h post-TBI (GSE290150) to characterize the landscape of immune and vascular cell populations during the acute phase and to identify candidate PVM–endothelial interaction pathways. We then employed BMDMs as a PVM surrogate model and conducted *in vitro* validation experiments using bEnd.3 cerebral endothelial cells to assess the role of MIF–ACKR3–STAT3 signaling in mediating endothelial activation–associated molecular changes. Our findings collectively support a candidate PVM-derived MIF–ACKR3 paracrine signaling axis that promotes endothelial stress activation and barrier-associated molecular alterations in the acute TBI setting.

## 2 Materials and methods

### 2.1 Single-cell RNA sequencing data source and preprocessing

**FIGURE 1:**
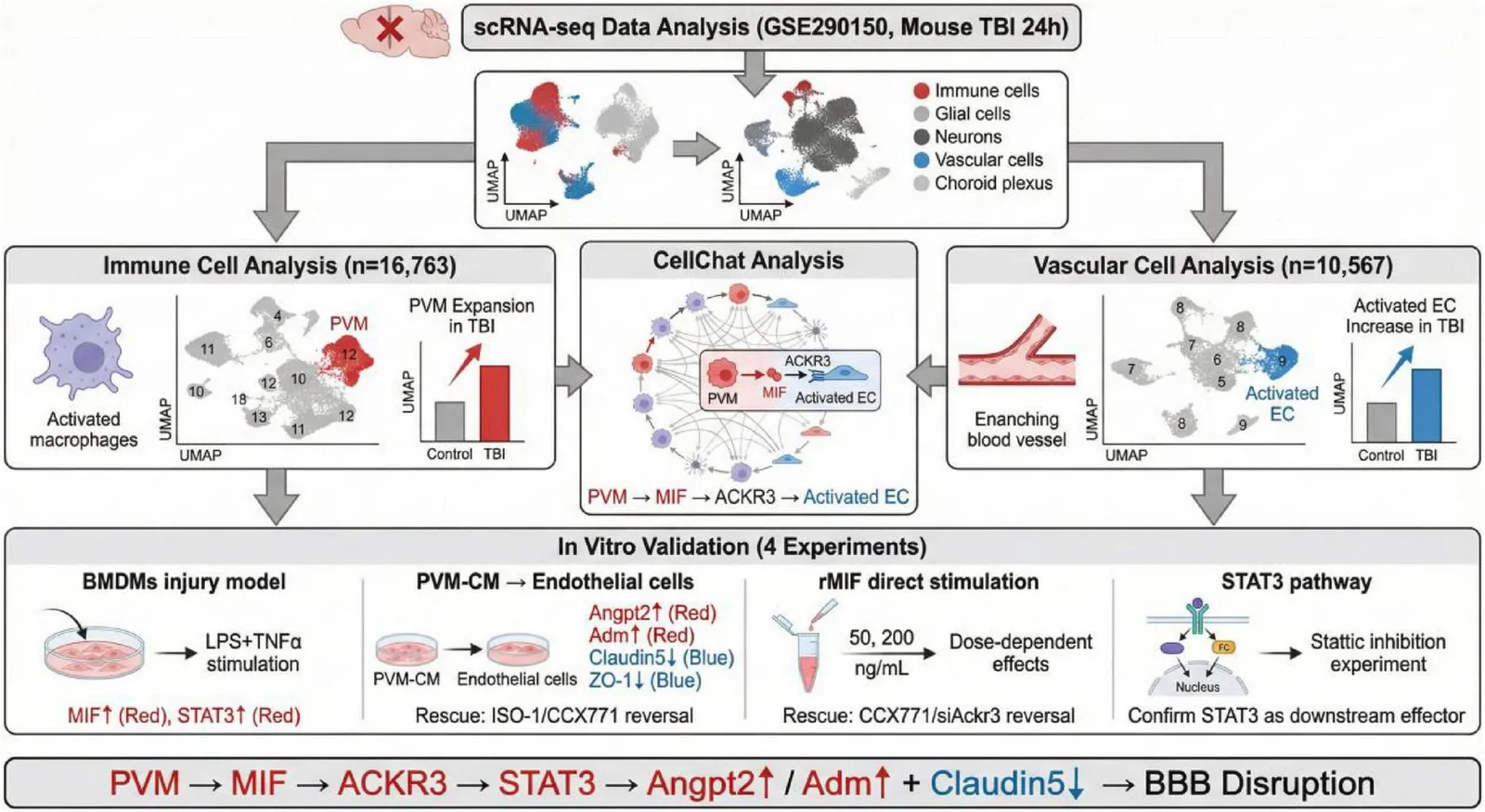
Schematic overview of the study design and analytical workflow. The study proceeded through four sequential stages: (1) quality control, data integration, clustering, and cell-type annotation of the mouse scRNA-seq dataset GSE290150 (24 h post-TBI); (2) sub-clustering of immune and vascular cell populations, cell composition differential analysis, and CellChat-based intercellular communication inference; (3) *in vitro* validation of macrophage MIF expression and secretion using the BMDM inflammatory injury model; and (4) assessment of MIF–ACKR3–STAT3 signaling in bEnd.3 cerebral endothelial cells using macrophage-conditioned medium and recombinant MIF stimulation paradigms. scRNA-seq, single-cell RNA sequencing; BMDM, bone marrow–derived macrophage; PVM, perivascular macrophage; MIF, macrophage migration inhibitory factor; ACKR3, atypical chemokine receptor 3; TBI, traumatic brain injury.

Figure 1 illustrates the overall methodological framework of this study. The scRNA-seq dataset GSE290150 was downloaded from the Gene Expression Omnibus (GEO) database. This dataset comprises brain tissue samples from mice subjected to controlled cortical impact (CCI)-induced TBI and sham surgery controls, collected 24 h post-injury. Raw count matrices were imported and analyzed using the Seurat R package (v4.3.0) (Hao et al., 2021). Quality control criteria were applied as follows: cells with 200–6,000 detected genes, mitochondrial gene fraction <15%, and UMI count > 1,000 were retained for downstream analysis. Doublet detection and removal were performed using the DoubletFinder package (McGinnis et al., 2019) to minimize artifactual cell multiplets.

### 2.2 Data Integration, dimensionality reduction, and clustering

Batch effects across samples were corrected using the Harmony algorithm (Korsunsky et al., 2019). Principal component analysis (PCA) was applied to the batch-corrected expression data, and the top 30 principal components were selected for UMAP-based dimensionality reduction and visualization. Graph-based clustering was performed using the FindNeighbors and FindClusters functions in Seurat at a resolution of 0.8. Clustering resolutions across a tested range (0.4–1.2) were compared with respect to cluster stability, canonical marker separation, and avoidance of over-fragmentation; resolution 0.8 provided the best balance for downstream biological interpretation. A clustering resolution sensitivity summary is provided in Supplementary Table 1. Major cell type annotation was based on canonical marker gene expression: immune cells (Ptprc), astrocytes (Aldh1l1), oligodendrocytes (Mbp), neurons (Rbfox3), endothelial cells (Pecam1), and pericytes (Pdgfrb).

### 2.3 Immune cell and vascular cell sub-clustering

Immune cells and vascular-related cells were separately extracted and subjected to independent sub-clustering analyses. Immune cell subpopulations were annotated based on the following canonical marker genes: T cells (Cd3e), homeostatic microglia (Tmem119, P2ry12), interferon-responsive microglia (Ifit1, Isg15), phagocytic microglia (Cd68, Lgals3), border-associated macrophages (BAMs; Ms4a7, Mrc1), and a PVM-like subpopulation (Cd163, Lyve1). Given the inherent resolution limitations of scRNA-seq in precisely delineating brain border macrophage subsets based on transcriptional features alone—particularly without spatial, ontogenetic, or flow cytometric validation, and in line with recent reviews emphasizing the need for multimodal delineation of brain macrophage compartments—this Cd163^+^Lyve1^+^ population is consistently referred to as the “PVM-like cell cluster” throughout the manuscript and should not be interpreted as an anatomically confirmed cell identity. Vascular cell subpopulation annotation referenced marker genes for capillary endothelial cells (Slco1a4), arterial endothelial cells (Bmx), activated endothelial cells (Vcam1, Icam1), pericytes (Rgs5), and vascular smooth muscle cells (Acta2).

### 2.4 Cell abundance differential analysis

Differences in cell composition between the sham and TBI groups were evaluated using the propeller method implemented in the speckle R package (Phipson et al., 2022). This statistical framework accounts for sample-level cell proportions and inter-sample variability in scRNA-seq compositional data, providing more robust estimates of cell type abundance changes than simple proportion comparisons. *P* < 0.05 was considered statistically significant. For comparisons involving multiple cell types, adjusted *P*-values were preferentially reported.

Gene-set scoring was performed using Seurat’s AddModuleScore function (default parameters). Inflammatory response scores were derived from the MSigDB Hallmark gene set “HALLMARK_INFLAMMATORY_RESPONSE.” Vascular regulation/remodeling-associated scores were computed from a curated union of GO biological process terms “vascular process in circulatory system,” “angiogenesis,” and “regulation of angiogenesis,” after removal of redundant genes. Module scores were compared between Sham and TBI within the indicated clusters using two-sided Wilcoxon rank-sum tests.

### 2.5 Cell–cell communication analysis

Candidate intercellular communication networks were inferred using the CellChat R package (v1.6.0) (Jin et al., 2021). Separate communication networks were constructed for the sham and TBI groups, and differential signaling pathway strengths were compared between conditions. Analyses were focused on candidate interactions between PVM-like cells and endothelial cell subpopulations, prioritizing pathways demonstrating enhanced communication strength in the TBI group. It is important to note that CellChat inferences are entirely computational and represent candidate interactions based on co-expression of curated ligand–receptor pairs; they do not constitute direct experimental evidence of functional paracrine signaling. To assess the robustness of CellChat-prioritized interactions, we additionally performed an orthogonal cell–cell communication analysis using the LIANA framework. Ligand–receptor pairs were ranked using LIANA consensus prioritization, and interactions involving PVM-like cells as senders and endothelial subpopulations as receivers were compared with CellChat outputs.

### 2.6 Cell culture

The mouse brain microvascular endothelial cell line bEnd.3 and 8-week-old male C57BL/6J mice were obtained from China ATCC repository. All mice were euthanized before femoral and tibial bone marrow harvest. Bone marrow cells were filtered and cultured in complete RPMI-1640 medium supplemented with 20 ng/mL macrophage colony-stimulating factor (M-CSF; PeproTech, 315-02) for 7 days to yield differentiated mature bone marrow–derived macrophages (BMDMs). BMDMs were used as a surrogate model for PVM-like cells under inflammatory conditions.

### 2.7 Experimental design and treatments

Macrophage inflammatory injury model: BMDMs were divided into three groups: Control; Injury (100 ng/mL LPS + 20 ng/mL TNFα, 24 h); and Injury + Stattic (5 μM STAT3 inhibitor Stattic, applied concurrently with inflammatory stimulation). This combined LPS + TNFα paradigm was selected to simulate the complex injury-associated inflammatory microenvironment of acute TBI rather than to model classical macrophage polarization states, and does not fully recapitulate *in vivo* TBI conditions. We selected this stimulus combination as a reproducible and experimentally tractable inflammatory challenge that robustly induces macrophage activation and MIF production for mechanistic testing of the candidate pathway. We agree that it more closely models a generalized inflammatory state than a sterile TBI-specific alarmin environment, and therefore interpret the BMDM findings as hypothesis-supporting rather than as a faithful recreation of *in vivo* post-traumatic macrophage activation.

Macrophage-conditioned medium (Mφ-CM) treatment of endothelial cells: Culture supernatants were collected from Injury-group BMDMs at 24 h, clarified by centrifugation (300 × *g*, 5 min), and diluted 1:1 with fresh DMEM/10% FBS for application to bEnd.3 cells over 24 h. Experimental groups comprised: Control (regular medium), Mφ-CM, Mφ-CM + ISO-1 (100 μM MIF inhibitor), and Mφ-CM + CCX771 (100 nM ACKR3 antagonist). Inhibitors were added to bEnd.3 cells at the commencement of the conditioned medium treatment phase.

Recombinant MIF stimulation: bEnd.3 cells were treated with recombinant mouse MIF (rMIF; R&D Systems) at 0, 50, or 200 ng/mL for 24 h. Additional groups comprised: rMIF (200 ng/mL) + CCX771 (100 nM) and rMIF (200 ng/mL) + siAckr3 (Ackr3-targeting siRNA). siRNA transfection was performed 48 h prior to rMIF stimulation. Ackr3 knockdown efficiency was verified at the transcript level by RT-qPCR; protein-level validation was not performed in the present study.

STAT3 pathway validation: bEnd.3 cells were divided into four groups: Control, rMIF (200 ng/mL), rMIF + Stattic (5 μM), and Stattic-only control. Stattic was applied 1 h prior to and maintained throughout rMIF stimulation.

### 2.8 Real-time quantitative PCR (rT-qPCR)

Total RNA was extracted using TRIzol reagent (Invitrogen) according to the manufacturer’s instructions. Complementary DNA (cDNA) synthesis was performed using the PrimeScript RT Reagent Kit (Takara). Quantitative PCR amplification was conducted using a SYBR Green detection system under the following thermal cycling conditions: initial denaturation at 95 °C for 30 s, followed by 40 cycles of 95 °C for 5 s and 60 °C for 34 s. Gapdh served as the internal reference gene, and relative expression was calculated using the 2^–ΔΔCT^ method. All primer sequences are provided in Supplementary Table 2.

### 2.9 Enzyme-linked immunosorbent assay (ELISA)

Culture supernatants were collected at designated time points and centrifuged to remove cellular debris. Protein concentrations of MIF (R&D Systems, DY1978), TNFα (R&D Systems, DY410), Angpt2 (R&D Systems, MANG20), and Adm (CUSABIO) were determined using commercial ELISA kits in strict accordance with each manufacturer’s instructions. Absorbance readings were obtained using a microplate reader, and protein concentrations were interpolated from standard curves.

### 2.10 Statistical analysis

All cell culture experiments were independently repeated four times (*n* = 4 biological replicates). For BMDM experiments, each biological replicate represents an independent bone marrow isolation and macrophage differentiation experiment. For bEnd.3 experiments, each biological replicate represents an independent treatment experiment performed on separate culture occasions/passages rather than technical replicate wells from the same plate. Data are presented as mean ± standard deviation (SD). Comparisons between two independent groups were analyzed using Student’s unpaired *t*-test; comparisons among three or more groups were assessed by one-way analysis of variance (ANOVA) followed by Tukey’s multiple comparisons *post-hoc* test. *P* < 0.05 was considered statistically significant. All statistical analyses and figure preparation were performed using R software (v4.2.0) and GraphPad Prism (v9.0).

## 3 Results

### 3.1 Single-cell transcriptional atlas of mouse brain tissue 24 h post-TBI

**FIGURE 2:**
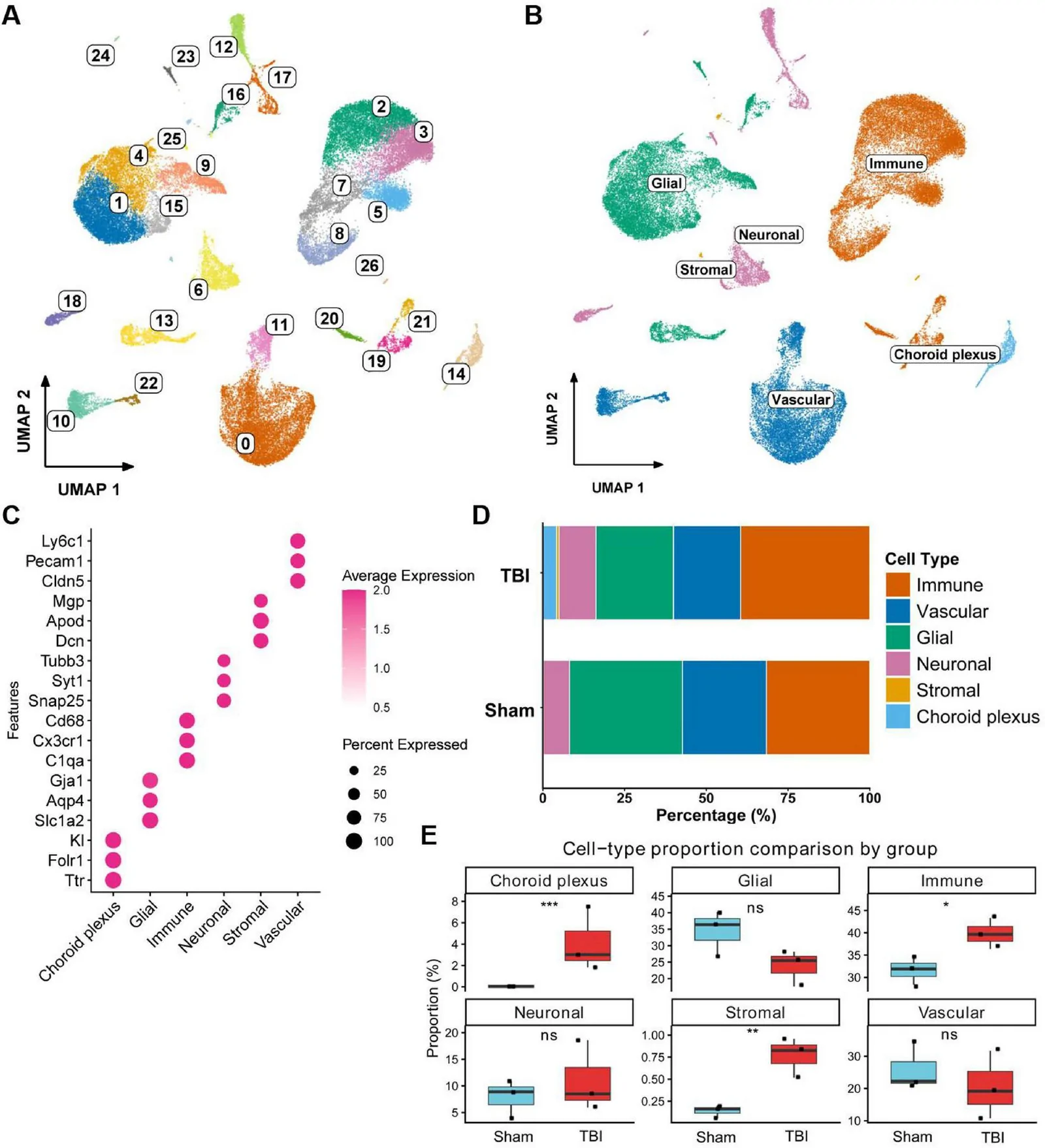
Single-cell transcriptomic landscape of brain tissue at 24 h after traumatic brain injury (TBI) and changes in major cell-type composition. **(A)** UMAP visualization of all 47,403 cells after quality control, batch correction, and clustering, revealing 27 cell clusters. **(B)** UMAPs annotated by major cell types, including immune cells, glial cells, neurons, vascular-associated cells, stromal cells, and choroid plexus–related cells. **(C)** Dot plots of representative marker gene expression across major cell types to support cell-type annotation. In dot plots, dot size indicates the percentage of cells within each cluster expressing the indicated gene, and color intensity indicates the scaled average expression level. **(D)** Comparison of major cell-type composition proportions between the Sham and TBI groups. **(E)** Propeller-based analysis of cell-type composition differences, showing a statistically significant increase in the proportion of immune cells in the TBI group.TBI, traumatic brain injury; UMAP, uniform manifold approximation and projection. *p < 0.05, **p < 0.01, ***p < 0.001.

Following stringent quality control filtering and doublet removal, 47,403 high-quality cells from the GSE290150 dataset were retained for downstream analysis. After Harmony-based integration and batch correction, PCA and graph-based clustering partitioned cells into 27 subclusters (Figure 2A). Based on canonical marker gene expression profiles, these subclusters were annotated into six major cell categories: immune cells, glial cells, neurons, stromal cells, vascular cells, and choroid plexus cells (Figures 2B, C). Comparison of major cell-type composition between sham and TBI groups revealed a pronounced increase in the proportion of immune cells in TBI animals (Figure 2D). Propeller-based statistical analysis confirmed a significant increase in immune cell abundance in the TBI group relative to sham controls (*P* < 0.01; Figure 2E), indicative of robust acute-phase immune remodeling within the injured brain.

### 3.2 Immune cell sub-clustering reveals significant PVM-like cell expansion after TBI

**FIGURE 3:**
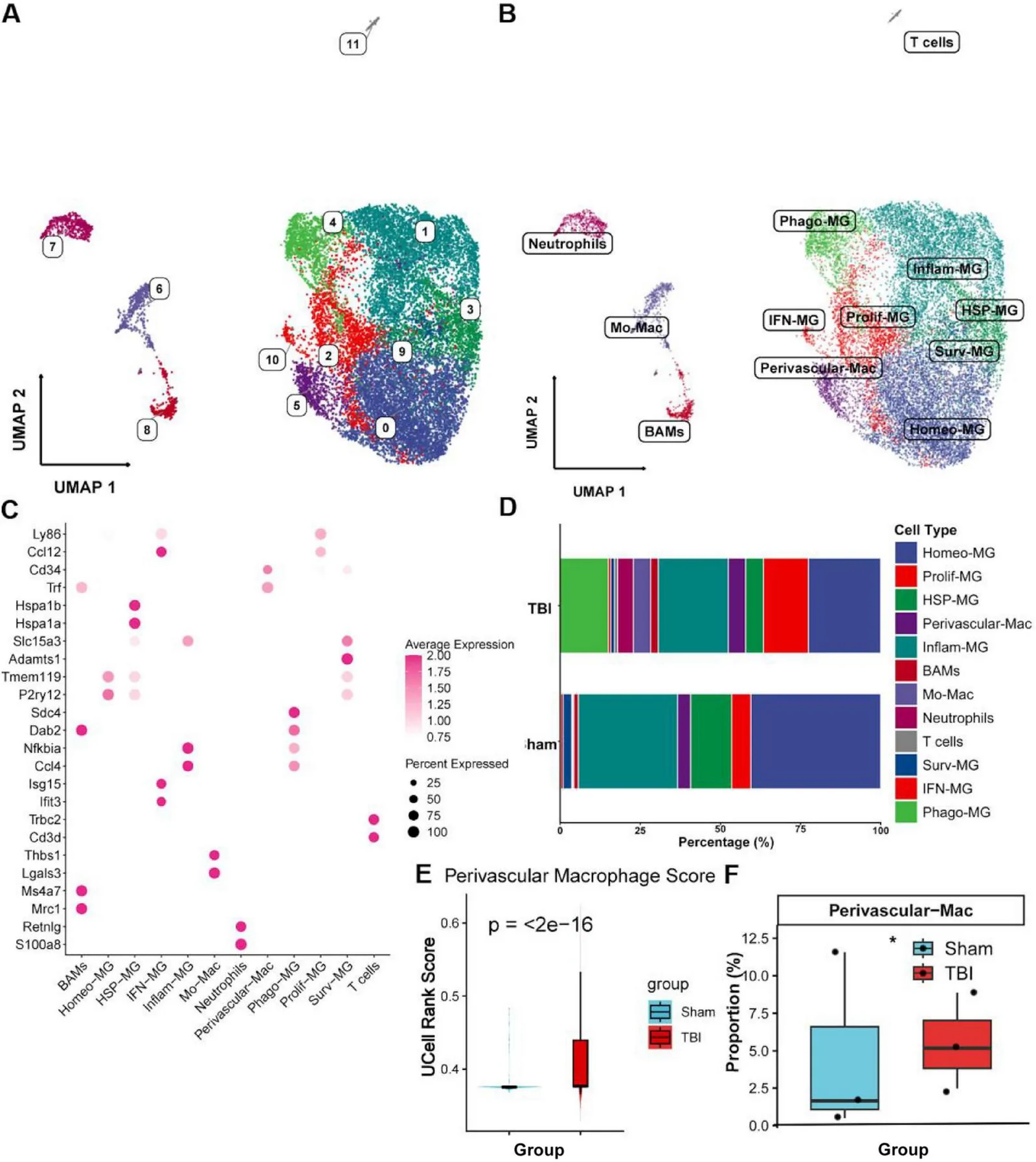
Immune cell subpopulation analysis reveals an increase in PVM-like cells after TBI. **(A)** UMAP of immune cells after secondary clustering, identifying 12 subpopulations. **(B)** Annotation of immune cell subpopulations, including T cells, microglia in various functional states, border-associated macrophages, and PVM-like cell clusters. **(C)** Expression patterns of representative marker genes across immune cell subpopulations to support annotation. In dot plots, dot size indicates the percentage of cells within each cluster expressing the indicated gene, and color intensity indicates the scaled average expression level. **(D)** Comparison of immune cell subpopulation proportions between Sham and TBI groups. **(E)** Propeller-based analysis of subpopulation composition, showing a statistically significant increase in the proportion of PVM-like cells in the TBI group. **(F)** Comparison of inflammation- and vascular-regulation–related gene-set scores between Sham and TBI for PVM-like cells. In boxplots, the center line denotes the median, the box indicates the interquartile range, and the whiskers extend to 1.5 × IQR. PVM, perivascular macrophage; BAM, border-associated macrophage; IFN-MG, interferon-responsive microglia; Phago-MG, phagocytic microglia. *p < 0.05.

To resolve immune cell heterogeneity at higher resolution, 16,763 immune cells were extracted and subjected to independent sub-clustering analysis, yielding 12 distinct subpopulations (Figures 3A, B). Based on canonical and subtype-specific marker gene expression, these subpopulations were annotated as: T cells (Cd3e^+^), homeostatic microglia (Tmem119^+^P2ry12^+^), interferon-responsive microglia (IFN-MG; Ifit1^+^Isg15^+^), phagocytic microglia (Phago-MG; Cd68^+^Lgals3^+^), BAMs (Ms4a7^+^Mrc1^+^), and a PVM-like subpopulation (Cd163^+^Lyve1^+^) (Figure 3C). Cell proportion comparisons revealed increased proportions of IFN-MG, Phago-MG, BAMs, and PVM-like cells in the TBI group relative to sham controls (Figure 3D). Propeller analysis confirmed that the expansion of PVM-like cells was statistically significant (*P* < 0.01; Figure 3E). Furthermore, gene set scoring revealed that PVM-like cells in the TBI group exhibited significantly elevated scores for inflammatory response and vascular regulation gene programs compared to sham controls (Figure 3F), suggesting that this population adopts an activated transcriptional state with potential vascular modulatory functions in the acute post-TBI period.

### 3.3 Vascular cell sub-clustering reveals enrichment of activated endothelial cells after TBI

**FIGURE 4:**
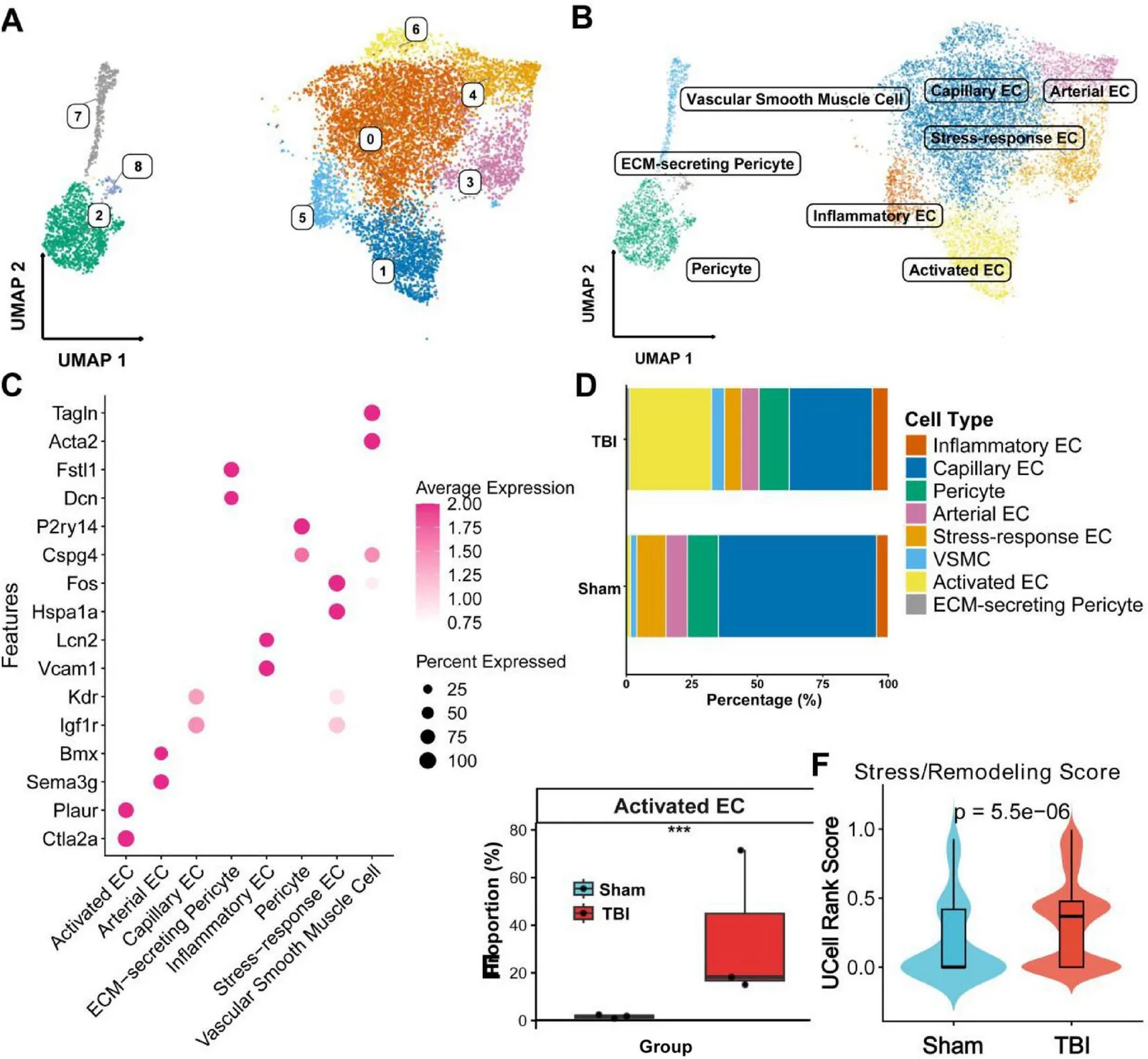
Vascular-related cell subpopulations reveal enrichment of activated endothelial cells after TBI. **(A)** UMAP of vascular-related cells after secondary clustering, identifying nine subpopulations. **(B)** Annotation of vascular-related cell subpopulations, including capillary, arterial, inflammation-associated, stress-responsive, and activated endothelial cells, as well as pericytes, ECM-secreting pericytes, and vascular smooth muscle cells. **(C)** Expression patterns of representative marker genes across vascular-related subpopulations.In dot plots, dot size indicates the percentage of cells within each cluster expressing the indicated gene, and color intensity indicates the scaled average expression level. **(D)** Comparison of vascular-related subpopulation proportions between Sham and TBI groups. **(E)** Propeller-based analysis of cell composition, showing a statistically significant increase in the proportion of activated endothelial cells in the TBI group. **(F)** Comparison of inflammation- and remodeling-related gene-set scores between Sham and TBI for vascular-related cells. EC, endothelial cell; ECM, extracellular matrix; VSMC, vascular smooth muscle cell. ***p < 0.001.

A total of 10,567 vascular-related cells were extracted for sub-clustering analysis, identifying nine distinct subpopulations (Figure 4A). These were annotated based on marker gene expression as: capillary endothelial cells (Slco1a4^+^), arterial endothelial cells (Bmx^+^), inflammatory ECs, stress-response ECs, activated ECs (Vcam1^+^Icam1^+^), pericytes (Rgs5^+^), ECM-secreting pericytes, and vascular smooth muscle cells (VSMCs; Acta2^+^) (Figures 4B, C). Cell abundance analysis revealed a pronounced increase in the proportion of Activated ECs in the TBI group (Figure 4D), which was statistically confirmed by propeller analysis (*P* < 0.01; Figure 4E). Functional gene set scoring additionally demonstrated enhanced inflammatory response and remodeling-associated gene programs in TBI vascular cells compared to sham controls (Figure 4F), indicating significant reactive reprogramming of the cerebral endothelium during the acute post-injury phase.

### 3.4 Cell–cell communication analysis predicts enhanced MIF–ACKR3 signaling between PVM-like cells and activated ECs after TBI

**FIGURE 5:**
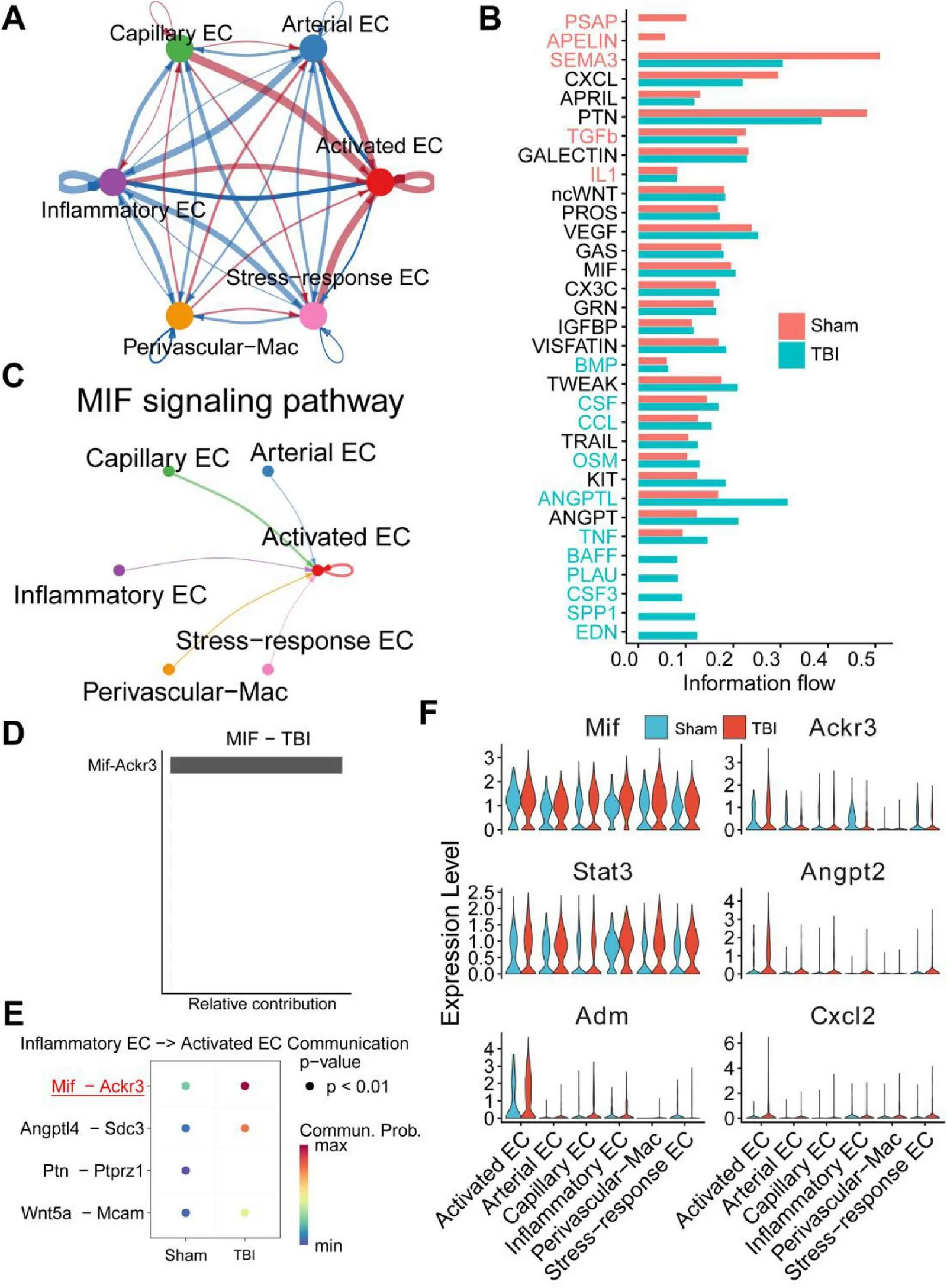
Cell–cell communication analysis predicts enhanced MIF–ACKR3 signaling between PVM-like cells and activated endothelial cells after TBI. **(A)** Overall cell–cell communication network inferred by CellChat, highlighting active signaling exchanges between PVM-like cells and endothelial cell subtypes. **(B)** Bubble plots of signaling pathways showing augmented strength in the TBI relative to Sham group, including MIF, CSF3, SPP1, and EDN-related pathways. **(C)** Sender–receiver pattern analysis for the MIF signaling pathway. **(D)** Visualization of MIF ligand–receptor interactions predicted from PVM-like cells to activated ECs. **(E)** Comparison of inferred MIF–ACKR3 communication strength between Sham and TBI for the PVM-like cell–Activated EC pair. **(F)** Visualization of expression levels of MIF in PVM-like cells and ACKR3, ANGPT2, ADM, and CXCL2 in Activated ECs across Sham and TBI conditions. CellChat results reflect inferred intercellular communications from transcriptomic co-expression data; they do not constitute direct evidence of functional receptor–ligand interactions *in vivo*. ACKR3, atypical chemokine receptor 3; PVM, perivascular macrophage; EC, endothelial cell.

To explore candidate intercellular communication between the expanded PVM-like population and activated endothelial cells, CellChat-based analysis was performed separately for the sham and TBI groups. Active candidate signaling exchanges were predicted between PVM-like cells and multiple endothelial subpopulations (Figure 5A). Compared to the sham group, several signaling pathways predicted to originate from PVM-like cells toward endothelial cells showed enhanced strength in TBI, including pathways related to CSF3, SPP1, EDN, and MIF (Figure 5B). Focused analysis of the MIF signaling pathway identified ACKR3 as the candidate receptor through which Activated ECs may receive MIF signals from PVM-like cells, with markedly elevated predicted communication strength in the TBI group relative to sham (Figures 5C–E). Ligand–receptor expression analysis confirmed significant upregulation of Mif and Stat3 in TBI PVM-like cells, and elevated Ackr3, Angpt2, and Adm in TBI Activated ECs; Cxcl2 showed minimal change (Figure 5F). These computational results designate the MIF–ACKR3 axis as a high-priority candidate communication pathway for subsequent experimental investigation. As an orthogonal computational check, LIANA-based analysis similarly prioritized the MIF–ACKR3 interaction among the leading PVM-like cell-to-Activated EC candidate pathways in the TBI group (Supplementary Table 3), supporting the robustness of the CellChat-based prioritization across inference frameworks. All CellChat outputs represent inferred communications derived from transcriptomic co-expression data and do not constitute direct biochemical or functional evidence of receptor–ligand interactions *in vivo*.

### 3.5 Macrophage MIF expression and secretion are upregulated under injury-simulating inflammatory conditions

**FIGURE 6:**
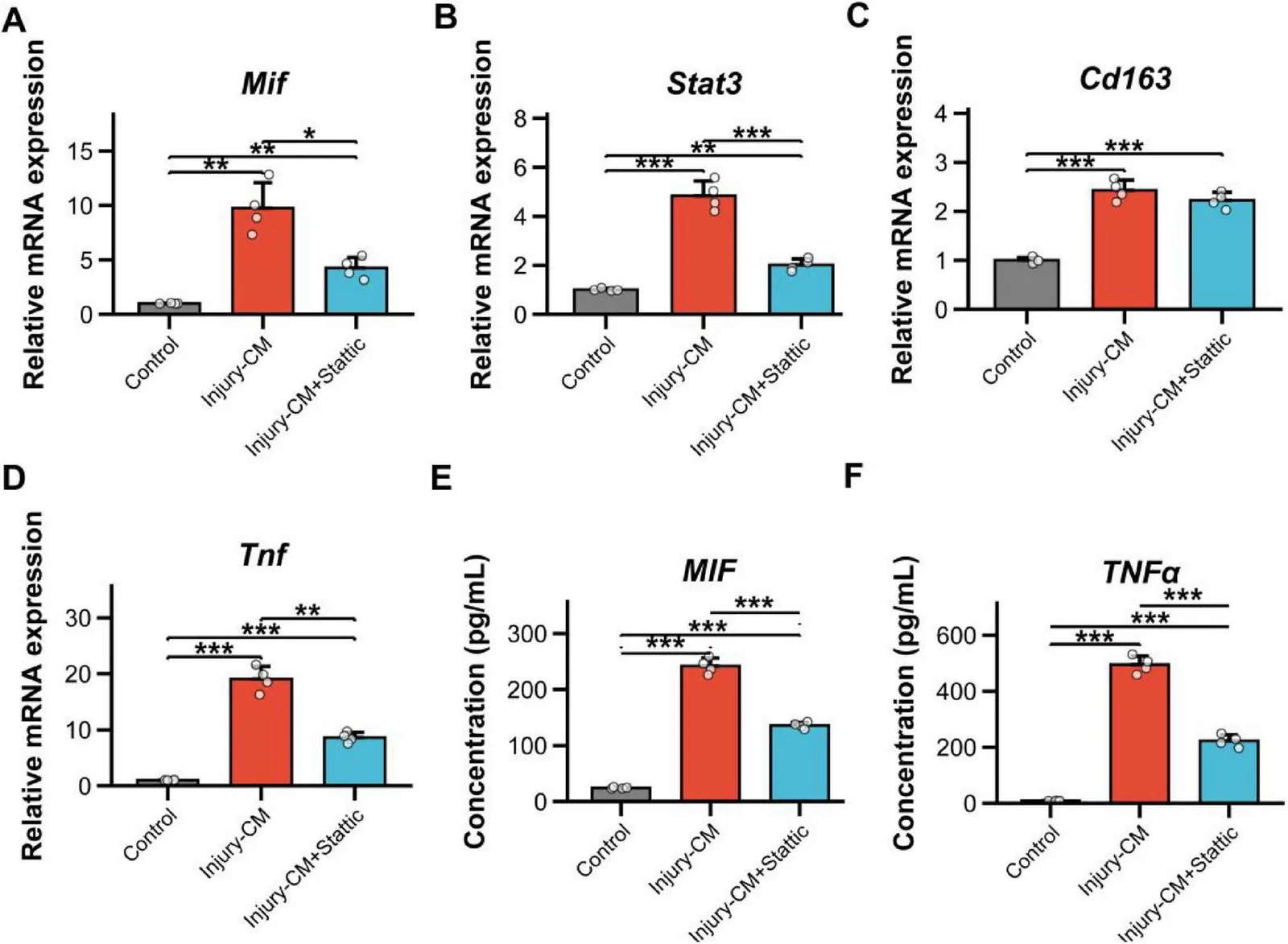
MIF expression and secretion are elevated in bone marrow–derived macrophages (BMDMs) under inflammatory injury-mimicking conditions, and are partially suppressed by STAT3 inhibition. BMDMs were divided into three groups: Control; Injury (100 ng/mL LPS + 20 ng/mL TNFα for 24 h); and Injury + Stattic (5 μM Stattic). **(A–D)** Relative mRNA expression levels of Mif, Stat3, Cd163, and Tnf measured by RT-qPCR. **(E,F)** Protein levels of MIF and TNFα in culture supernatants measured by ELISA. Data are shown as mean ± SD (*n* = 4 biological replicates). Statistical analysis was performed using one-way ANOVA followed by Tukey’s *post-hoc* test. *P* < 0.05, *P* < 0.01, *P* < 0.001. BMDM, bone marrow–derived macrophage. Relative mRNA levels were normalized to Gapdh and calculated by the 2^–ΔΔCT^ method. *p < 0.05, **p < 0.01, ***p < 0.001.

To validate candidate PVM-derived MIF signaling identified by single-cell analysis, we established an *in vitro* macrophage inflammatory activation model using BMDMs. Compared to controls, LPS + TNFα treatment for 24 h significantly elevated Mif mRNA expression in BMDMs (9.76 ± 2.31-fold, *P* < 0.001) alongside markedly increased Stat3 transcript levels (4.84 ± 0.58-fold, *P* < 0.001) (Figures 6A, B). The macrophage marker Cd163 and inflammatory cytokine Tnf were also substantially upregulated (Figures 6C, D). ELISA confirmed significant increases in both MIF and TNFα protein concentrations in culture supernatants of stimulated BMDMs (Figures 6E, F). Addition of the STAT3 inhibitor Stattic partially but significantly reduced Mif mRNA expression and MIF protein secretion (*P* < 0.01), indicating that STAT3-related transcriptional activity contributes to the regulation of macrophage MIF production under these injury-simulating conditions. The concurrent upregulation of Mif and Stat3 observed *in vitro* mirrors the transcriptional pattern identified in TBI PVM-like cells by single-cell analysis.

### 3.6 Macrophage-conditioned medium induces endothelial activation–associated molecular changes attenuated by MIF–ACKR3 blockade

**FIGURE 7:**
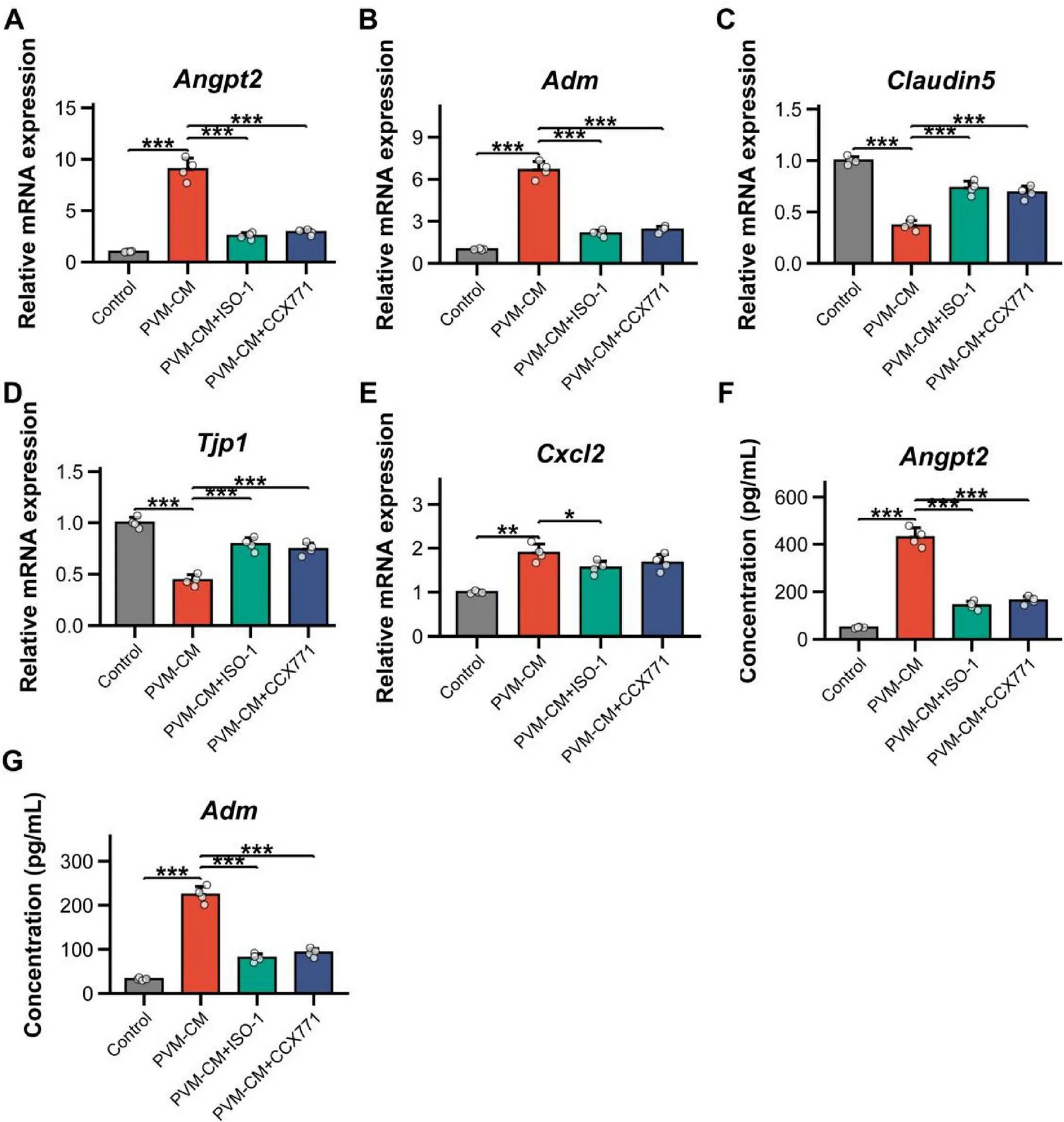
Macrophage-conditioned medium induces endothelial activation–associated molecular changes and downregulates tight junction–associated genes in bEnd.3 cells; effects are attenuated by MIF inhibition or ACKR3 blockade. **(A–E)** Relative mRNA expression levels of Angpt2, Adm, Claudin5, Tjp1, and Cxcl2 in bEnd.3 cells measured by RT-qPCR. **(F,G)** Protein levels of Angpt2 and Adm in culture supernatants measured by ELISA. Data are presented as mean ± SD (*n* = 4 biological replicates). Statistical analysis was performed using one-way ANOVA followed by Tukey’s *post-hoc* test. *P* < 0.05, *P* < 0.01, *P* < 0.001. Mφ-CM, macrophage-conditioned medium; ISO-1, MIF inhibitor; CCX771, ACKR3 antagonist. Relative mRNA levels were normalized to Gapdh and calculated by the 2^–ΔΔCT^ method. *p < 0.05, **p < 0.01, ***p < 0.001.

To assess the paracrine effects of macrophage-derived factors on cerebral endothelial biology, conditioned medium from inflammatorily activated BMDMs (Mφ-CM) was applied to bEnd.3 cells. ELISA measurement of the Injury-group BMDM supernatant showed a MIF concentration of 100 ng/mL before dilution. Because this conditioned medium was applied to bEnd.3 cells after 1:1 dilution with fresh medium, the approximate MIF exposure concentration during Mφ-CM treatment was 50 ng/mL, providing a reference point for comparison with the 50–200 ng/mL rMIF doses used in the direct stimulation experiments. Compared to control medium, Mφ-CM treatment significantly upregulated Angpt2 (9.02 ± 1.08-fold, *P* < 0.001) and Adm (6.66 ± 0.63-fold, *P* < 0.001) mRNA in bEnd.3 cells (Figures 7A, B), and concomitantly downregulated Claudin5 (0.37 ± 0.05-fold, *P* < 0.001) and Tjp1 (0.44 ± 0.05-fold, *P* < 0.001) (Figures 7C, D). MIF inhibitor ISO-1 (100 μM) and ACKR3 antagonist CCX771 (100 nM) both substantially attenuated Mφ-CM-induced Angpt2 and Adm upregulation and partially restored Claudin5 and Tjp1 expression (*P* < 0.01).

Because Claudin5 and Tjp1 were assessed only at the mRNA level in the present study, these findings should be interpreted as barrier-associated transcriptional alterations rather than direct evidence of tight junction protein loss or junctional disorganization. In contrast, the reaction of Cxcl2 with Mφ-CM showed only a slight increase (Figure 7E). ELISA further confirmed elevated Angpt2 and Adm protein levels in culture supernatants of Mφ-CM-treated bEnd.3 cells, which were significantly reduced by either ISO-1 or CCX771 treatment (Figures 7F, G). These results indicate that macrophage-derived soluble factors can induce endothelial activation–associated molecular changes at least partially through MIF–ACKR3-dependent mechanisms.

### 3.7 Recombinant MIF directly induces endothelial molecular changes in an ACKR3-dependent manner

**FIGURE 8:**
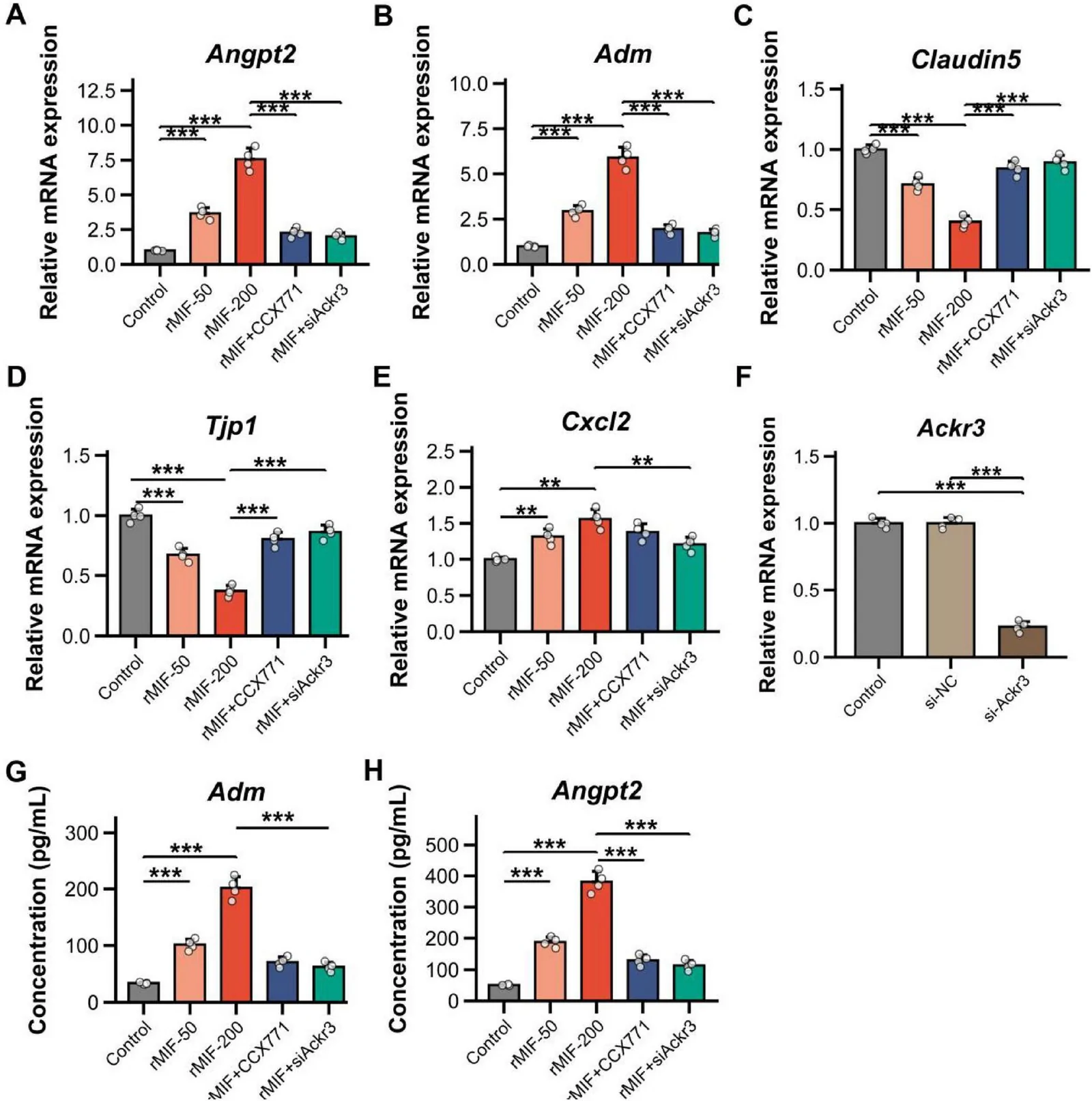
Recombinant mouse MIF induces dose-dependent molecular changes in bEnd.3 cells, and these effects are attenuated by ACKR3 pharmacological blockade or siRNA knockdown. **(A–E)** Relative mRNA expression levels of Angpt2, Adm, Claudin5, Tjp1, and Cxcl2 in bEnd.3 cells measured by RT-qPCR. **(F)** Validation of Ackr3 knockdown efficiency by RT-qPCR following siRNA transfection. **(G,H)** Protein levels of Angpt2 and Adm in culture supernatants measured by ELISA. Data are presented as mean ± SD (*n* = 4 biological replicates). Statistical analysis was performed using one-way ANOVA followed by Tukey’s *post-hoc* test. *P* < 0.05, *P* < 0.01, *P* < 0.001. rMIF, recombinant mouse MIF; siAckr3, Ackr3-targeting siRNA; CCX771, ACKR3 antagonist. Relative mRNA levels were normalized to Gapdh and calculated by the 2^–ΔΔCT^ method. **p < 0.01, ***p < 0.001.

To isolate the specific contribution of MIF to the endothelial changes observed with conditioned medium and to minimize confounding from other macrophage-derived mediators, bEnd.3 cells were directly stimulated with recombinant mouse MIF (rMIF). rMIF induced dose-dependent upregulation of Angpt2 and Adm: at 50 ng/mL, fold increases reached 3.67 ± 0.41 and 2.94 ± 0.31, respectively; at 200 ng/mL, fold increases reached 7.57 ± 0.79 and 5.90 ± 0.59 (all *P* < 0.001; Figures 8A, B). Concomitant downregulation of Claudin5 and Tjp1 was observed at both doses (Figures 8C, D). Cxcl2 showed only modest induction; where statistically detectable, effect sizes remained small relative to Angpt2 and Adm and were not consistently reproduced across CM and rMIF paradigms (Figure 8E). Pharmacological ACKR3 blockade with CCX771 or siRNA-mediated Ackr3 knockdown (knockdown efficiency validated at the transcript level in Figure 8F) significantly attenuated rMIF-induced Angpt2 and Adm upregulation and partially restored Claudin5 and Tjp1 expression. Although protein-level ACKR3 knockdown was not assessed in the present study, transcript-level suppression was sufficient to blunt the downstream endothelial molecular phenotype. ELISA confirmed significantly reduced Angpt2 and Adm protein secretion with CCX771 or siAckr3 treatment (Figures 8G, H). These results demonstrate that MIF can directly drive endothelial activation–related molecular changes in an at least partially ACKR3-dependent manner. As in the conditioned medium experiments, the Claudin5 and Tjp1 readouts in this section are transcript-level measurements and therefore indicate molecular barrier-associated changes rather than direct proof of altered tight junction protein abundance or localization.

### 3.8 STAT3 signaling mediates downstream endothelial responses to MIF–ACKR3 activation

**FIGURE 9:**
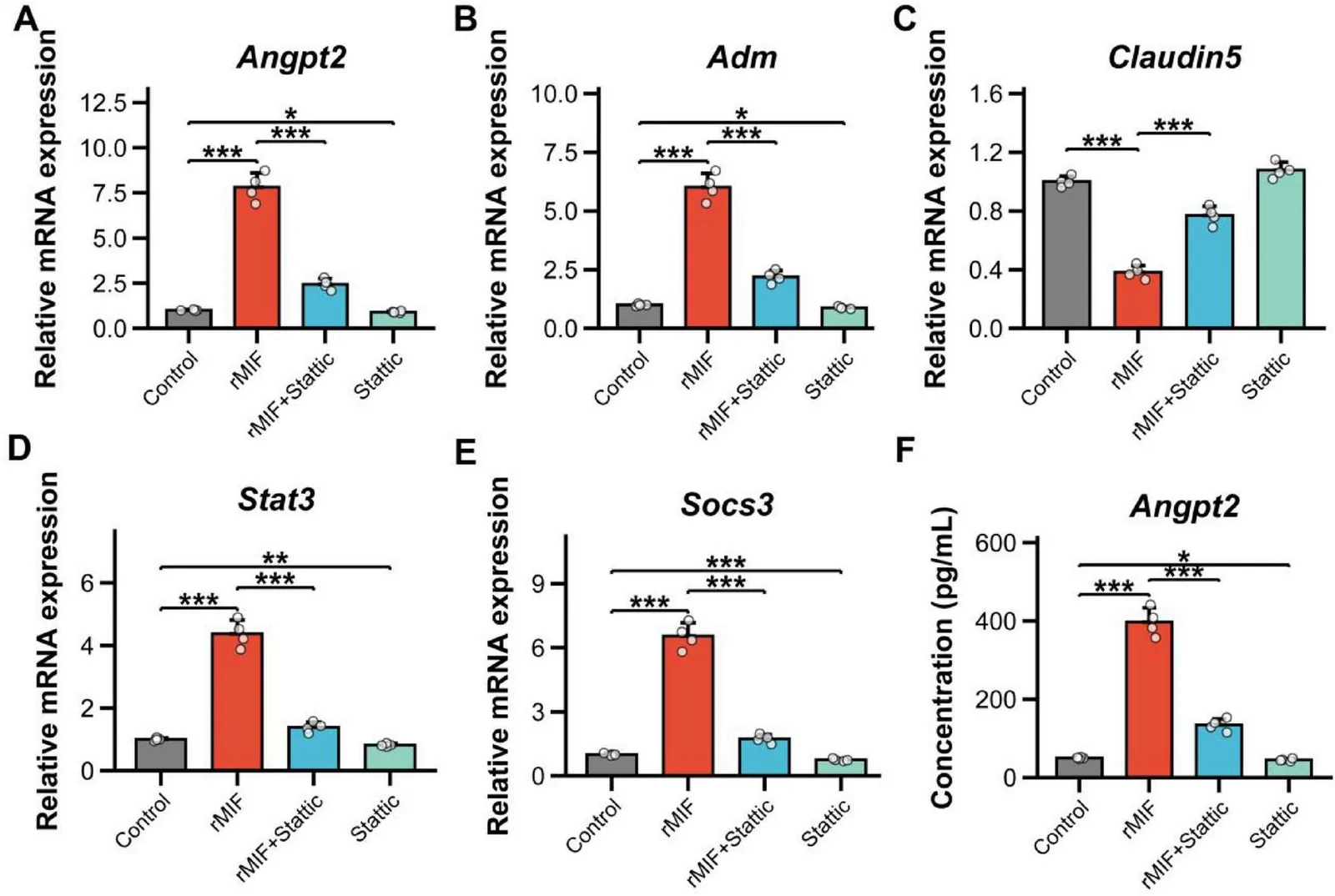
STAT3 inhibition attenuates MIF-induced endothelial activation–related molecular changes in bEnd.3 cells. bEnd.3 cells were treated with rMIF (200 ng/mL) with or without the STAT3 inhibitor Stattic (5 μM). **(A–C)** Relative mRNA expression levels of Angpt2, Adm, and Claudin5 measured by RT-qPCR. **(D,E)** Expression changes of Stat3 and Socs3 to assess STAT3-related transcriptional activity measured by RT-qPCR. **(F)** Angpt2 protein concentration in culture supernatants measured by ELISA. Data are presented as mean ± SD (*n* = 4 biological replicates). Statistical analysis was performed using one-way ANOVA followed by Tukey’s *post-hoc* test. *P* < 0.05, *P* < 0.01, *P* < 0.001. rMIF, recombinant mouse MIF; Stattic, STAT3 pathway inhibitor. Relative mRNA levels were normalized to Gapdh and calculated by the 2^–ΔΔCT^ method. *p < 0.05, **p < 0.01, ***p < 0.001.

To delineate the intracellular signaling cascade downstream of MIF–ACKR3 engagement in endothelial cells, bEnd.3 cells were treated with rMIF in the presence or absence of the STAT3 inhibitor Stattic. Stattic significantly suppressed rMIF-induced Angpt2 and Adm mRNA upregulation and partially restored Claudin5 expression compared to rMIF treatment alone (Figures 9A–C). At the transcriptional level, rMIF elevated both Stat3 and Socs3 mRNA levels, and Stattic attenuated these changes (Figures 9D, E). ELISA additionally confirmed that Stattic significantly reduced rMIF-induced Angpt2 protein secretion (Figure 9F). The Stattic-only control group showed no significant changes in any measured parameter relative to untreated controls, supporting the specificity of the observed inhibitory effects. These results identify STAT3-related signaling as a downstream intracellular mediator of the MIF-induced endothelial activation–associated molecular program in bEnd.3 cells.

## 4 Discussion

This study integrated single-cell transcriptomic analysis of acute TBI brain tissue with *in vitro* macrophage and endothelial cell experiments to characterize candidate mechanisms of immune–vascular crosstalk in the acute post-injury period. The principal findings are as follows: (1) scRNA-seq analysis of brain tissue 24 h post-TBI revealed concurrent expansion of a PVM-like macrophage population and Activated ECs, both exhibiting injury-associated transcriptional reprogramming; (2) CellChat-predicted intercellular communication indicated enhanced MIF–ACKR3 signaling from PVM-like cells toward Activated ECs in TBI; (3) inflammatory stimulation of BMDM surrogates robustly upregulated MIF production in a STAT3-dependent manner; (4) macrophage-conditioned medium and recombinant MIF induced endothelial activation–associated molecular changes—Angpt2/Adm upregulation and Claudin5/Tjp1 downregulation—through ACKR3-dependent mechanisms; and (5) STAT3 inhibition suppressed MIF-induced endothelial transcriptional responses. Taken together, these findings support a candidate PVM-derived MIF–ACKR3–STAT3 signaling axis contributing to endothelial activation and barrier-associated molecular alterations in acute TBI.

### 4.1 PVMs as candidate active modulators of acute neurovascular injury in TBI

Perivascular macrophages are brain-resident macrophages occupying the perivascular Virchow–Robin spaces along penetrating cerebral vessels, developmentally and functionally distinct from parenchymal microglia (Kierdorf et al., 2019; Lapenna et al., 2018). Under homeostatic conditions, PVMs maintain cerebrovascular integrity through immune surveillance, regulation of vascular tone, and facilitation of metabolic waste clearance via the glymphatic system (Drieu et al., 2022). Notably, PVMs have been implicated in neurovascular dysfunction across multiple disease contexts: Faraco et al. (2016) demonstrated that PVM depletion ameliorates hypertension-induced cognitive impairment and neurovascular coupling deficits, while Hawkes and McLaurin (2009) showed that PVM depletion reduces perivascular amyloid clearance in cerebral amyloid angiopathy. PVMs have also been reported to respond dynamically to ischemic vascular injury, transitioning from homeostatic surveillants to reactive inflammatory effectors (Pedragosa et al., 2018).

In the present study, PVM-like cell proportions were significantly elevated at 24 h post-TBI relative to sham controls, and these cells exhibited enhanced inflammatory response and vascular regulation gene program scores. This activation pattern is consistent with the concept that cerebrovascular-associated macrophages contribute to early neurovascular remodeling following acute injury (Jassam et al., 2017; Simon et al., 2017). However, we must explicitly acknowledge that the current study utilized a scRNA-seq-based cell cluster defined by Cd163/Lyve1 marker co-expression, which represents a PVM-associated transcriptional signature rather than an anatomically confirmed perivascular identity. As emphasized by recent reviews of brain macrophage diversity, precise separation of perivascular macrophages from meningeal macrophages and related BAM subsets requires complementary spatial, ontogenetic, and immunophenotypic validation. Without complementary immunofluorescence localization, parabiosis experiments to assess turnover dynamics, or fate-mapping approaches, the precise ontogenetic and anatomical identity of this cluster cannot be fully established. Future studies employing spatial transcriptomics or immunohistochemical co-localization with established perivascular markers would importantly advance the characterization of this population in the TBI context.

### 4.2 The MIF–ACKR3 axis as a candidate PVM-to-endothelial paracrine pathway

Migration inhibitory factor was originally characterized as a factor that inhibits macrophage migration but is now recognized as a pleiotropic cytokine with broad roles in inflammation and immune regulation (Calandra and Roger, 2003). Structurally, MIF engages distinct receptor complexes: the CD74/CD44 complex mediates canonical ERK1/2 and PI3K/Akt signaling (Leng et al., 2003), while ACKR3 has been identified as a non-cognate but functionally relevant MIF receptor capable of mediating β-arrestin-biased intracellular signaling (Alampour-Rajabi et al., 2015; Bernhagen et al., 2007). In vascular biology, MIF has been implicated in endothelial inflammation, monocyte recruitment to the vessel wall, and atherosclerotic plaque progression (Zernecke et al., 2008); its role specifically at the cerebrovascular interface in acute TBI has been less characterized.

Our CellChat analysis predicted the MIF–ACKR3 pair as a significantly upregulated candidate communication pathway between PVM-like cells and Activated ECs in TBI. This prediction was supported by convergent transcriptional evidence: Mif and Stat3 were upregulated in TBI PVM-like cells, while Ackr3, Angpt2, and Adm were upregulated in TBI Activated ECs. The subsequent *in vitro* experiments provided direct mechanistic support for this axis: both conditioned medium and recombinant MIF induced consistent endothelial molecular changes that were substantially attenuated by ACKR3 blockade or knockdown. The appropriate epistemic framing must be maintained: CellChat-inferred interactions represent computational predictions, not proven *in vivo* paracrine events, and the *in vitro* system cannot fully recapitulate the complexity of the post-TBI neurovascular microenvironment.

Atypical chemokine receptor 3 is expressed on cerebral endothelial cells and regulates multiple aspects of vascular biology including chemokine scavenging (Naumann et al., 2010) and angiogenic signaling (Hattermann et al., 2010). Our data show that Ackr3 is transcriptionally upregulated in Activated ECs post-TBI, which may represent a mechanism for enhanced endothelial sensitivity to macrophage-derived MIF signals in the injured brain. It is also important to note that ISO-1 functions as a MIF enzymatic tautomerase inhibitor and acts upstream of all MIF receptor pathways; therefore, the attenuation of conditioned medium effects by ISO-1 alone cannot be attributed exclusively to ACKR3-mediated signaling, and the relative contributions of CD74 versus ACKR3 remain to be fully delineated.

### 4.3 MIF-induced endothelial molecular alterations and their relevance to BBB biology

The endothelial molecular response profile induced by macrophage-conditioned medium and recombinant MIF in the present study—upregulation of Angpt2 and Adm alongside downregulation of Claudin5 and Tjp1—is consistent with an endothelial activation and barrier-associated molecular alteration state. Downregulation of Claudin-5 and ZO-1 (Tjp1) at the mRNA level is a transcriptional pattern reported in inflammatory and injury-associated endothelial contexts, and is concordant with the single-cell finding of elevated Vcam1 and Icam1 in the Activated EC cluster. However, it must be explicitly stated that the current study measured gene expression changes and secreted protein levels without directly quantifying monolayer permeability using functional assays such as transendothelial electrical resistance (TEER) measurement or tracer flux assays, and without assessing tight junction protein subcellular localization by immunofluorescence. The present data are therefore most accurately framed as evidence of “MIF-induced barrier-associated molecular alterations” rather than as direct demonstration of MIF-mediated BBB functional impairment. This distinction is scientifically important and should guide the interpretation of these findings.

Furthermore, bEnd.3 cells exhibit phenotypic differences from primary brain microvascular endothelial cells, including lower baseline tight junction protein expression and attenuated barrier function characteristics (Brown and Davis, 2002). This limitation should be considered when extrapolating these findings to the *in vivo* BBB context. Validation in primary human or mouse brain endothelial cells, or in more physiologically representative BBB-on-chip microfluidic models, would substantially strengthen the mechanistic conclusions.

### 4.4 Angpt2 and Adm as molecular indicators of MIF-driven endothelial stress

Angiopoietin-2 (Angpt2) is a key context-dependent regulator of vascular homeostasis: it destabilizes the endothelium by competing with Angpt1 for Tie2 receptor binding, and is consistently upregulated in inflammatory, infectious, and traumatic vascular injury states (Augustin et al., 2009; Scholz et al., 2015). Circulating Angpt2 levels have been proposed as biomarkers of endothelial activation in sepsis and other critical illness contexts (David et al., 2012), and its upregulation has been associated with vascular permeability increases in cerebrovascular disease. The pronounced Angpt2 upregulation induced by MIF-related stimuli in our system identifies it as a potentially important downstream effector through which the PVM–endothelial MIF–ACKR3 axis could contribute to vascular destabilization in acute TBI.

Adrenomedullin (Adm) exhibits complex, context-dependent vascular biology. Originally characterized as a potent vasodilatory peptide (Kitamura et al., 1993), Adm exerts primarily cytoprotective and barrier-stabilizing effects in certain inflammatory conditions, where it has been shown to reduce vascular hyperpermeability (Temmesfeld-Wollbrück et al., 2007). However, Adm is also a stress-inducible gene in endothelial cells responding to hypoxia, cytokine challenge, and mechanical stress, functioning as part of a broader vascular adaptive transcriptional response (Hinson et al., 2000). The upregulation of Adm in bEnd.3 cells following MIF stimulation in our study is therefore most conservatively interpreted as a component of an endothelial stress response and vascular regulatory transcriptional program, rather than as evidence of a specific directional functional outcome. Whether Adm upregulation in this context is ultimately cytoprotective or contributes to pathological vascular remodeling in TBI requires dedicated functional investigation.

### 4.5 Dual roles of STAT3 in the MIF–ACKR3 signaling axis

STAT3 is a pleiotropic transcription factor with established roles across immune, epithelial, and vascular cell types (Hillmer et al., 2016; Yu et al., 2014). Our study reveals an apparent dual function of STAT3 within the MIF–ACKR3 axis: at the macrophage (PVM-like cell) level, STAT3 promotes Mif transcription and MIF secretion, as evidenced by the capacity of Stattic to suppress Mif expression in stimulated BMDMs. The concurrent upregulation of Mif and Stat3 in TBI PVM-like cells in our scRNA-seq data is consistent with this regulatory relationship. At the endothelial cell level, STAT3 appears to function as a downstream intracellular effector of MIF signaling, mediating transcriptional induction of Angpt2 and Adm while contributing to suppression of tight junction gene expression. The elevation of Socs3—a canonical STAT3 transcriptional target serving as a negative feedback regulator—provides indirect transcriptional evidence of STAT3 pathway engagement in endothelial cells following rMIF stimulation. This dual-compartment role is conceptually consistent with the pleiotropic functions of STAT3 across cell types, and with prior evidence that STAT3 can regulate Angpt2 promoter activity (Fiedler et al., 2006) and modulate claudin family gene expression (Shen et al., 2019).

However, several important caveats apply: the present evidence for STAT3 involvement is based entirely on pharmacological inhibition using Stattic and mRNA-level transcriptional readouts, without protein phosphorylation (p-STAT3) measurements, chromatin immunoprecipitation (ChIP) assays, or genetic loss-of-function approaches. Furthermore, Stattic may have off-target effects beyond STAT3 inhibition. Definitive mechanistic conclusions regarding STAT3’s specific role in mediating MIF–ACKR3 endothelial signaling will therefore require additional biochemical and molecular validation in future studies. This caveat is particularly important because ACKR3 is widely described as a β-arrestin-biased receptor, and our current data do not distinguish canonical JAK/STAT3 activation from indirect or non-canonical signaling routes. Direct assessment of p-STAT3 (Tyr705), together with analysis of β-arrestin- and ERK-related signaling, will be necessary to resolve the intracellular signaling architecture downstream of MIF–ACKR3 engagement in brain endothelial cells.

### 4.6 Minimal Cxcl2 induction suggests preferential endothelial reprogramming over chemokine amplification

The consistently minimal and non-significant Cxcl2 upregulation observed across both conditioned medium and recombinant MIF stimulation experiments warrants specific discussion. Cxcl2 encodes a potent neutrophil-attracting CXC chemokine whose robust induction would be expected if MIF were primarily driving a classical pro-inflammatory, leukocyte-recruitment endothelial response. The relative selectivity of MIF-induced endothelial changes toward Angpt2/Adm upregulation and Claudin5/Tjp1 downregulation—rather than chemokine amplification—suggests that the predominant endothelial response in this context may reflect vascular state reprogramming toward an activated, barrier-compromised molecular phenotype, rather than classical inflammatory chemokine amplification. This pattern is conceptually consistent with a model in which endothelial barrier molecular alterations may precede and facilitate subsequent inflammatory cell recruitment during the early post-TBI period (Engelhardt et al., 2017). However, this interpretation is speculative and requires validation through combined temporal *in vivo* analyses. It should also be noted that the CD74 receptor pathway for MIF was not specifically assessed in the current study, and the relative contributions of CD74- versus ACKR3-mediated signaling to the observed endothelial responses remain to be delineated through receptor-specific loss-of-function approaches.

### 4.7 Study limitations and future directions

Several important limitations of this study must be acknowledged. First, the transcriptomic component of this study is based on re-analysis of a single public scRNA-seq dataset obtained at one post-injury timepoint (24 h) in one experimental model (CCI). Accordingly, the present analysis captures only a single snapshot of a highly dynamic pathophysiological process and should not be generalized to the full acute-to-subacute TBI trajectory or to the heterogeneity of human TBI. We additionally reviewed the recently released CCI atlas GSE269748 at the level of published annotations and overall cellular trends; although differences in sampling strategy, preprocessing, and annotation granularity precluded direct pooled analysis, its reported early border-associated macrophage activation and endothelial inflammatory reprogramming are directionally consistent with our observations. Nevertheless, formal cross-dataset validation of the specific PVM-like–Activated EC MIF–ACKR3 interaction remains an important future step. Second, BMDMs were employed as a PVM surrogate model and do not fully recapitulate the functional state, transcriptional profile, or brain-specific microenvironmental adaptations of resident brain perivascular macrophages; findings should therefore be interpreted as mechanistic hypotheses rather than as direct evidence of PVM function *in vivo*. Third, bEnd.3 cells exhibit phenotypic differences from primary brain microvascular endothelial cells with respect to tight junction expression, barrier properties, and receptor expression profiles; validation in primary cells or more physiologically relevant models is warranted. Fourth, the study assessed endothelial responses exclusively at the mRNA and secreted protein levels, without direct measurement of barrier permeability (TEER, FITC-dextran flux), tight junction protein localization by immunofluorescence, or *in vivo* BBB leakage measurements. Fifth, STAT3 involvement was assessed pharmacologically and at the transcript level, without phospho-protein (p-STAT3) analysis, ChIP assays, or genetic loss-of-function approaches. Sixth, contributions of alternative MIF receptors (CD74, CXCR4) to the observed endothelial responses were not systematically excluded. Seventh, Ackr3 knockdown was verified at the mRNA rather than protein level, and future studies incorporating receptor protein quantification would further strengthen receptor-specific mechanistic interpretation.

Future studies addressing these limitations should: (1) employ immunofluorescence co-staining or spatial transcriptomics to verify perivascular localization of MIF-expressing macrophages post-TBI; (2) use conditional macrophage-specific Mif knockout models or PVM depletion strategies to assess the *in vivo* contribution of PVM-derived MIF to BBB disruption; (3) measure TEER, tracer flux, or plasma protein extravasation to directly evaluate the effect of MIF–ACKR3 axis modulation on barrier function *in vitro* and *in vivo*; (4) assess p-STAT3 protein levels and perform ChIP assays to mechanistically confirm STAT3 as a downstream transcriptional effector; (5) use primary human or mouse brain microvascular endothelial cells or organotypic BBB models to validate key findings; and (6) investigate associations between MIF–ACKR3 molecular signatures and TBI severity or clinical outcomes in patient cohorts.

## 5 Conclusion

Through integrated single-cell transcriptomic analysis and *in vitro* mechanistic experiments, this study provides evidence supporting concurrent expansion and activation of PVM-like macrophages and Activated ECs in the acute post-TBI brain, with predicted enhancement of MIF–ACKR3-directed paracrine communication between these populations. *In vitro* experiments further demonstrate that inflammatory stimulation of macrophage surrogates promotes MIF production via STAT3-related mechanisms, and that macrophage-derived MIF induces endothelial activation–associated molecular changes—including Angpt2 and Adm upregulation alongside Claudin5 and Tjp1 downregulation—in cerebral endothelial cells through ACKR3- and STAT3-dependent signaling. These findings propose the MIF–ACKR3–STAT3 axis as a candidate regulatory pathway contributing to endothelial activation and barrier-associated molecular alterations in acute TBI, highlighting perivascular immune–vascular crosstalk as a potential avenue for therapeutic investigation. The direct *in vivo* contribution of this axis to functional BBB impairment and its translational relevance require validation in more complex experimental models and patient-derived systems.

## Data Availability

The raw data supporting the conclusions of this article will be made available by the authors, without undue reservation.
